# The role of dopamine in the life history of bivalves: a review

**DOI:** 10.1186/s12983-026-00607-4

**Published:** 2026-03-30

**Authors:** Viktoria Nikishchenko, Vyacheslav Dyachuk

**Affiliations:** https://ror.org/05t43vz03 grid.417808.2 0000 0001 1393 1398 A.V. Zhirmunsky National Scientific Center of Marine Biology, Far Eastern Branch, Russian Academy of Sciences, Vladivostok, Russia

**Keywords:** Bivalves, Mollusks, Larvae, Dopamine, Development, Nervous system

## Abstract

Dopamine is one of the best-known neurotransmitters found in most animals. Among the major roles that this mediator plays in many organisms is the regulation of motor skills, behavior, and feeding. Its localization in the central (CNS) and peripheral nervous systems (PNS) has been well studied mainly in vertebrate animals, but remains poorly understood in invertebrates such as mollusks of the class Bivalvia. Nevertheless, bivalves are of particular interest for their nervous system that has undergone a number of simplifications due to the sedentary lifestyle. As shown in the review, the key functions of dopamine have been retained in this group, with, however, a shift towards regulation of effector organs and physiological processes. The interaction between serotonin and dopamine, which regulates the degree of motor activity, nutrition, and locomotion, also deserves special consideration. There still remains a vast number of unresolved issues concerning the effects that dopamine exerts in the bivalve CNS, its role in the regulation of larval development and behavior of adults. This review summarizes the major known aspects of dopamine, including its localization and role in the life history of bivalves.

## Introduction

**Fig. 1:**
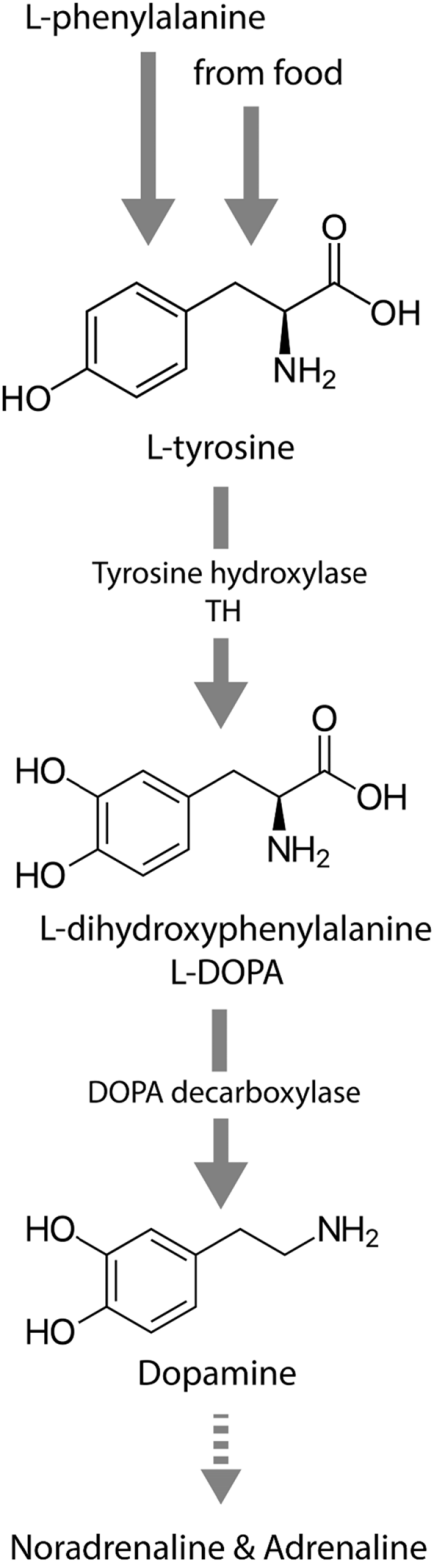
Diagram of DA synthesis

Dopamine (DA) is a neurotransmitter belonging to the group of monoamines that are derivatives from an aromatic amino acid containing one amino group in the molecule [1]. It is also classified into the group of catecholamines (CA) including adrenaline (epinephrine) and noradrenaline (norepinephrine). All of these neurotransmitters are derivatives of L-tyrosine (which, in turn, can either be taken up from the environment or be synthesized from phenylalanine) and are produced through the same process as CA biosynthesis. In general, the DA synthesis occurs as follows (Fig. 1): a hydroxyl group is attached to the L-tyrosine aromatic ring by the enzyme tyrosine hydroxylase, thus, forming L-dihydroxyphenylalanine (L-DOPA); then the amino group is decarboxylated by DOPA decarboxylase; and, as a result, DA is synthesized. Noradrenaline is formed from DA through hydroxylation, and adrenaline is formed from noradrenaline through methylation.

All CA perform neurohumoral and neurotransmitter functions. In particular, DA is known as the “feel-good” hormone responsible for regulating such a complex brain process as the reward system, or internal reinforcement: behavior regulation and control based on positive reinforcement of actions [2]. The second most important and well-known function of DA is the regulation of motor activity. In mammals, one of the largest clusters of DAergic neurons is found in the substantia nigra (SN) located in the midbrain and spinal cord [3]. Damage to the SN and death of DAergic neurons in it causes a neurodegenerative disorder known as Parkinson’s disease, manifested as impaired motor inhibition, with such symptoms as limb tremor, hypokinesia, muscle rigidity, autonomic and mental disorders [4]. In addition to its neurotransmitter function, DA is a hormone regulating vascular pressure and heart rate, for which it is often used in emergency care as a milder cardiac resuscitating agent compared to adrenaline [5]. Furthermore, DA has an effect on digestive tract motility [6].

All the above-mentioned DA functions have been comprehensively studied in vertebrates, in particular mammals exhibiting higher nervous activity, but invertebrates have not been considered in as much detail. As regards bivalves, DA is known to regulate mainly motor activities such as locomotion and feeding [7]. In larval stages, DA along with serotonin, another monoamine, is involved in the regulation of larval behavior and tropism [8]. However, all bivalve studies have focused largely on members of the subclass Autobranchia, leaving aside the subclass Protobranchia and freshwater bivalves. All these findings indicate the importance of DA and provide a vast field for research to elucidate its role in the life history of bivalves, which can substantially contribute to the knowledge in such areas as aquaculture, neurobiology, and ecology.

## Historical background

The first studies on CA were conducted as early as in the late 19th and early twentieth centuries when Oliver and Schäfer [9, 10] published a number of papers on the biologically active, vasopressive effects caused by extracts of the calf suprarenal capsules. DA (then known as beta-3,4-hydroxyphenylethylamine) was first synthesized in 1910 by Barger and Ewins at the Wellcome Research Laboratories in London [11]. Later on, Barger and Dale found that DA had an epinephrine-like effect [12]. In the scientific community, this compound did not attract due attention and was long neglected by researchers. In 1939, Holtz with co-authors discovered the biogenic synthesis of DA (described as oxytyramine) from L-DOPA in renal tissues of guinea pigs [13]. Blaschko [14] hypothesized that oxytyramine might be an intermediate link in the biosynthesis of adrenaline and noradrenaline that had been well known by then.

Throughout the first half of the twentieth century, the main biological role of DA was largely a mystery to scientists. Traces of it were found in peripheral tissues and body fluids, and it was reported to have a weak, adrenaline-like effect on blood vessels of the body. By the 1950s, assumptions were made about the possible unique functions of DA that should differ from those of other CA. A study by Montagu that showed the presence of DA in the brain of mammals, including humans, gave new impetus to addressing this issue [15]. It was soon followed by a study of Carlsson with co-authors that focused on the neurotransmitter function of DA [16]. In 1958, Carlsson and Waldeck described a fluorimetric method for determining the presence and amount of DA in tissues [17]. Due to his significant efforts in this field of research and for his study on DA as a neurotransmitter and its association with Parkinson’s disease, Carlsson was awarded the Nobel Prize in 2000 [18].

A crucial moment in the study of CAergic systems, especially those in invertebrates, was the invention of methods for fluorescent imaging of CA-containing structures. The first approaches were based on the Pictet–Splenger reaction: frozen tissue sections were treated with hot formaldehyde vapors with subsequent condensation of CA into fluorescent compounds of dihydroisoquinolines. As a result, CA-containing structures emitted fluorescent glow that could be observed through a microscope [19, 20]. Subsequently, the methods were modified. Thus, it was discovered that glyoxylic acid forms chemical bonds with CA much faster and more specifically than formaldehyde, which made it possible to visualize tissue areas with a ten-fold lower level of CA than that required by the formaldehyde-based methods [20, 21]. Nevertheless, glyoxylic acid was considered not the best tissue fixative, which was especially important for a number of histological studies. Thus, the FaGlu method was proposed based on a mixture of formaldehyde and glutaraldehyde whose reaction with CA in tissues induced clearly observable fluorescence, while making it possible to cut histological sections of tissues on a vibratome and cryotome; drying of the treated tissue further enhanced the glow [20, 22]. The addition of sucrose to such a mixture even extended the potential of the FaGlu method, allowing visualization of CA-containing structures not only in histological sections but also in the whole body of animals, in particular mollusks [23].

The discovery of CA in vertebrate tissues inevitably raised the question of their presence in invertebrates as well. A vast number of experiments on cephalopods, gastropods, and bivalves to address the issue of adrenaline’s stimulating effect on the heart muscle were set up as early as in the 1930s and 1940s [24]. The first study on the presence of DA in bivalve tissues was published in 1963 by Sweeney who used a modified Carlsson’s fluorimetry method mentioned above [25]. The study also revealed a neurohumoral (i.e., neurotransmitter) role in regulating the life activity of bivalves. In the same year, another research team reported that serotonin (5-HT) and CA exerted a relaxing effect on the posterior adductor muscle in *Anodonta cygnea* [26]. The use of histofluorescence methods according to Falk and Hillarp [27] showed that DA and 5-HT in such mollusks as *Anodonta piscinalis*, *Helix pomatia*, and *Buccinum undatum* are largely localized in the CNS nervous tissue and also revealed a morphological and biochemical similarity of the monoaminergic neurons between the studied mollusks and vertebrates [28]. As was found later through differential and gradient centrifugation of the *Anodonta* nervous tissue, monoamines perform the function of interneuronal mediators [29]. The pharmacological effects of opioid drugs such as morphine, methionine-enkephalin, D-ala_2_-met-enkephalinamide, endorphin, and etorphine on the bivalve nervous system were also studied. Their effects on the DAergic system of bivalves indicated a functional relationship between opioids and the DAergic system [30–32].

Subsequent studies, mainly those by Drs. Aiello, Catapane, and Stefano, considered the localization of DA in tissues. The histofluorescence methods showed that DA in bivalves is predominantly localized in the gill nerves of the peripheral nervous system (PNS) [33, 34], in the gonads [35], and in the gut walls [36]. The major role of DA and 5-HT in regulating the beating of gill epithelium cilia and their opposing (antagonistic) effects to each other were clarified: the increased activity with exposure to 5-HT and, vice versa, the decreased activity with exposure to DA [37]. Similar antagonistic effects of DA and 5-HT were observed in experiments on the role of the pedal ganglion in controlling foot movements: the addition of 5-HT and DA caused the extension and contraction of the foot, respectively [38]. DA was later found in bivalve larvae [39, 40]. Then pharmacological experiments were conducted on larvae behavior and feeding [8]. To date, such issues as the tissue content, functions, and role of DA in development of bivalves have been considered only in few papers, because the research activity in this field increased largely in the late twentieth century. The present review is aimed to raise interest in investigating the role and function of DA in bivalves.

## Dopamine: identification, visualization, and mechanism of biosynthesis in bivalves

DA in bivalves, as well as in other animals, is synthesized from L-tyrosine (see Fig. 1), with the main site of synthesis being the CNS. Monoamines, DA precursors, are found in all ganglia. DA level depends on seasonal, temperature, and stress variations [30, 41–43]. As was shown by radiolabeling of monoamines including L-tyrosine, these are absorbed and synthesized both in perikaryons and neuropils of ganglia [44]. Besides, glial cells are also involved in the uptake of monoamines [45]. The direct transport of monoamines occurs through the axonal processes of commissures [45]. In the bivalve *Placopecten magellanicus*, a number of metabolites are formed through DA metabolism, indicating degradation of such CA as normetanephrine, metanephrine, 3,4-dihydroxyphenylacetic acid, and homovanillic acid that are found in vertebrates as well, which suggests that the CA biosynthesis mechanisms may be shared by most animals [46].

## Dopamine in adult bivalves: localization and functions

**Fig. 2:**
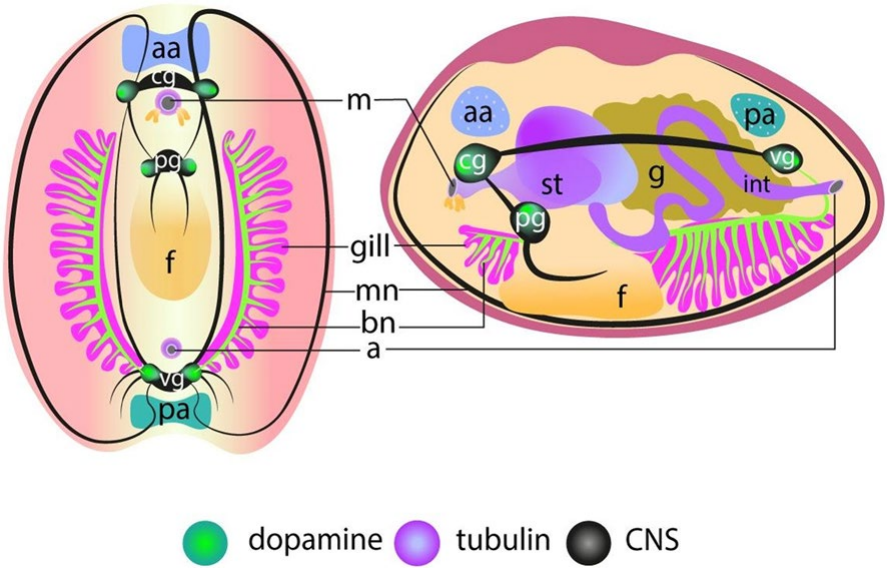
Anatomical organization of nervous system in adult bivalve mollusk. Color designations: green—DA; magenta—tubulin; black—central nervous system (CNS). Letter designations: a—anus; aa—anterior adductor; bn—branchial nerves; cg—cerebral ganglia; g—gonads; f—foot; int—intestines; m—mouth; mn—mantle; pa—posterior adductor; pg—pedal ganglia; st—stomach; vg—visceral ganglia

The localization of DA and its receptors in tissues and organs may somehow indicate its potential functions in animal physiology. The earliest studies on DA in bivalves focused on its presence in nervous tissues [25, 47]. A suggestion was made about the neurohumoral role of DA, which subsequently was confirmed. DA is present in all ganglia: cerebro-pleural, pedal, and visceral (Fig. 2) [7]. Besides the CNS, DA is also found in the PNS (Fig. 2), in particular the gill nerves, pedal and labial nerves, where the effect of DA is somehow and almost always associated with the regulation of motor, ciliary beating, and feeding activities [7].

In most cases, a double innervation by the DAergic and 5-HTergic systems is observed, with the functions of these systems being also antagonistic. 5-HT activates ciliary beating of the ciliated gill epithelium and foot extension, while DA reduces the ciliary beating rate up to ciliostasis and causes the foot to contract [37, 38]. Destruction of 5-HTergic nerves by 5,6-dihydroxytryptamine or DAergic nerves by 6-hydroxydopamine leads to the opposite effects, respectively [37, 38].

**Fig. 3:**
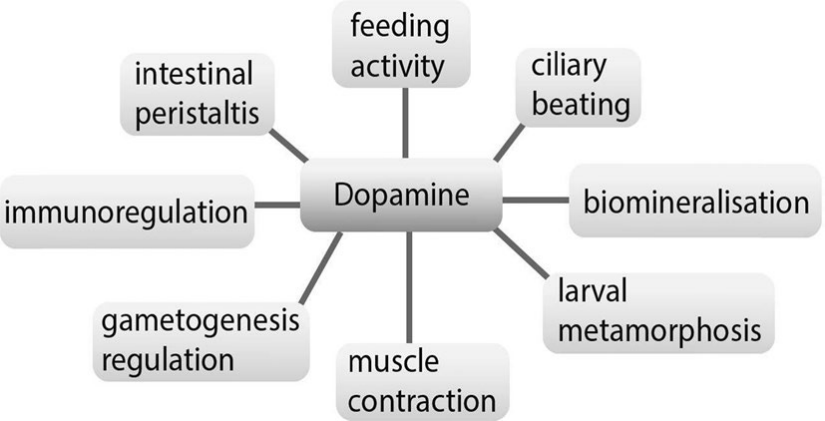
Peripheral regulatory roles of DA

Furthermore, the presence of DA in the bivalve gonads [35] was found to be associated with the regulation of oogenesis, maturation, and oocyte locomotion in the ovarian development [48] (Fig. 3). DA levels are also the highest in the female gonads at the proliferation stage, while minimum levels are recorded at the maturity stage [49].

The presence of DA nerves in the bivalve guts was shown by fluorescent histochemical methods [36]. In superfusion experiments, electrical stimulation of the rectum released both DA-like and acetylcholine-like substances. It was, therefore, assumed that DAergic nerves synapse with intestinal smooth muscle cells.

DA plays an important role in the physiology of adult bivalves, including crucial functions associated with feeding, movement, and respiration. The effect of DA injection on body excretion and heart rates indicates a relationship between DA levels and physiological parameters [50, 51]. DA in adult bivalves is often studied in the context of its interaction with other neuropeptides and hormones: (1) interaction of DA with FMRFamide and opioid peptides in regulating the catch mechanism of adductors [52]; (2) modulation of valve opening induced by DA, 5-HT, and the counteracting role of neuropeptide F [53]; (3) competing effects of DA and FMRFamide [54]; and (4) integration of 5-HT and DA into modulation of respiratory activity [37]. Thus, DA in adult bivalves plays several important roles that increase chances to survive and breed [55]. However, adult bivalves may be particularly vulnerable to dysfunction of the DA system, as evidenced by the excessive valve opening caused by low DA levels during development [56]. Since many bivalves are an important link in the biogeochemical cycle and are a food item for vertebrates, toxins that degrade biogenic amines can affect influential ecological groups. Therefore, understanding of how DA and other biogenic amines influence the bivalve’s behavior under stress conditions in ontogeny may help identify environmental problems caused by ongoing climate change.

Breeding in bivalves is regulated by seasonal and environmental factors, but little is known about the intrinsic factors influencing these processes. As in cases of other mollusks, very few studies have been conducted on the role of biogenic amines in the reproduction of bivalves. DA has been reported as a key regulator of male reproductive function for several invertebrate and vertebrate species [57, 58]. DA levels in gonadal tissues of marine bivalves vary throughout the breeding cycle. Incubation with DA caused oocytes to grow in a manner similar to that described from other species [35, 48].

The studies where DA content was measured in bivalve tissues did not reveal any seasonal fluctuations but showed a relationship with the reproductive strategy of bivalves: lower levels of DA indicated poor animal’s health, while high levels indicated energy stored in cells for the future rapid and high vitellogenesis when environmental conditions become more favorable [49]. The molecular bases of the neuroendocrine control of energy reserves and vitellogenesis in these organisms have not been sufficiently addressed to date.

The DA system is assumed to be involved in the control and coordination of the feeding behavior in bivalves (Fig. 3). A reduction in DA levels leads to relaxation of the catch-state due to a decrease in the electrical activity of the adductor and byssal retractor muscles in *Mytilus edulis* [59]. Nevertheless, excess DA causes a similar effect [60]. The role of DA in controlling the modulation of water processing in bivalves has also been described [51]. In general, the DAergic effects on feeding of bivalves suggest coordinated activity of this neurotransmitter in a classical behavior, at least in higher-order animals. The molecular mechanisms of this process have yet to be elucidated for better understanding of the DA system in this animal group.

Bivalves have survived all the environmental changes and mass extinctions. These animals exhibit excellent adaptability to various habitat conditions. However, we know substantially less about their endocrine and paracrine systems compared to those in other invertebrates. DA is the main molecule of the brain’s reward system which regulates behavioral processes such as arousal, motivation, learning, and memory [55, 61]. In mollusks, DA performs an inhibitory function by reducing the electron transport chain, oxygen consumption, and metabolic rate [51, 62, 63].

In bivalves, DA and other biogenic amines, since they emerged, have likely been involved in controlling the processes of digestion, absorption, storage, and transformation into other useful molecules, with their level regulating the release of digestive enzymes [8, 36, 63]. Other vitally important rapid reactions include the need for physical activity, feeding, growth, maturation, and breeding [61, 64–67].

DA in bivalves plays an immunoregulatory role in the inflammatory immune response where the nitric oxide (NO) production is used by the host to eliminate parasites [68, 69]. DA synthesis is reduced as bivalves enter the reproductive phase. It affects the breeding cycles by slowing down them [49, 70]. DA is also a key component of the body’s stress and immune system responses [68]. An increase in the expression of genes associated with CAergic immunomodulation pathways, especially the activity of such enzymes as DA beta-monooxygenase and monooxygenase, was observed in oyster larvae after exposure to heat and bacterial stress [71]. Therefore, DA acts as a precursor in the synthesis of adrenaline and noradrenaline that are involved in the regulation of hemocyte apoptosis through the adrenergic pathway [72].

Minimum concentrations of DA and a specific DA receptor were recorded from hemocytes of bivalves [73]. Moreover, some drugs that can influence or stimulate the immune system of vertebrates, when tested in vitro or in vivo on bivalve hemocytes, were found to change the parameters related to the presumptive interaction of DA receptors, thus, suggesting the possible presence of this receptor in bivalve cell lines [74, 75]. However, the preliminary data obtained by the use of specific agonists or antagonists of vertebrate DA receptors instead of DAergic genes to induce bivalve’s responses in vitro and/or to identify similarities with these genes sequence have not provided an unambiguous answer. Recently, a close similarity of DA receptor ligands in vertebrates and bivalves has been hypothesized [76]. Due to such receptors, CA showed a potential to enhance the activation of mollusk hemocytes when it was tested in its pure form, but not as an unpurified specimen [74].

The mussel shell development is based on the expression of genes and the deposition of calcium carbonate in the shell matrix protein secreted by the mantle cells. Thus, bivalves are of great interest for analyzing the effects of biogenic secretion of monoamines. The latter perform the mineralizing function to involve calmodulin (CaM), MAP kinases, integrin kinases, and aspartate and serine kinases in a positively regulated intracellular feedback signaling pathway for Ca^2+^/CaM, including K^+^ channels that release the content of chelated Ca^2+^/CaM providing Ca^2+^ activation [77–80]. DA can modulate gene transcription activity in almost all mussel’s cells through specific receptors and is considered an indicator of gene expression for Ca^2+^ channels, L-type Ca^2+^ channels, intracellular Ca^2+^ stores with voltage-dependent Ca^2+^ channels, and IP3/ryanodine receptors [81].

It has been shown that CgD1DR-1, a homolog of the DA D1 receptor found in bivalves, influences the expression of a number of genes responsible for the larval shell formation at the trochophore and D-veliger stages in *Crassostrea gigas* [82]*.* The blockage of CgD1DR-1 with an antagonist, SCH-23390 (halobenzazepine), slows down the expression of shell formation-related genes such as CgTyrosinase-1, CgTyrosinase-3, CgChitinaseLP, CgAMC, CgBMP, and CgBMPR. In addition, exposure to CO_2_ leads to the inhibition of DA synthesis and mRNA expression of CgD1DR-1, which indicates the vulnerability of bivalves to ocean acidification that is directly related to ongoing global warming.

## Dopamine receptors in bivalves

As known to date, bivalves have seven types of DA receptors (d1-like, d2-like, d3, d4, d5, d6, and d7) identified from several species [83]. A phylogenetic analysis has shown that d1-like and d2-like receptors originated before the split of bivalves and gastropods, while d4–d7 receptors appeared in pre-cephalopod lineages [83]. In general, DA receptors in bivalves are wider distributed over tissues than those in the entire phylum Mollusca but show evidently different distribution patterns between species. Bivalve d1-like and d2-like receptors share higher identity with those in gastropods than in polychaetes and cephalopods [84]. Also, d1-like and d2-like receptors in bivalves are relatively larger than those in gastropods. Moreover, bivalve DA receptors share unique features at the 2nd and 4th intracellular loops compared to those in other mollusks [83]. Similarly to bivalve d1-like receptors, bivalve d2-like receptors share specific amino acid deletions or substitutions compared to other mollusks [83]. The multiple alignments on the selected DA receptors indicate that bivalve d1-like and d2-like receptors also contain all the highly conserved domains essential for receptor activation. DA receptors have been implicated in various physiological roles including the modulation of locomotor rhythm, learning and memory, courtship, and heart rate [64, 65, 85]. For DA-mediated responses in *Amphioxus*, DA receptors (d1-like and d2-like) are sufficient to trigger the fast escape behavior [86]. Pharmacological manipulation of DA in bivalves have provided evidence to assume its involvement in the mediation of ciliary beating and adductor muscle contraction [52, 87]. Bivalve DA receptors have recently become a subject of pharmacological studies and transgenic receptor assays to explore the functional significance and ecological implications of the receptor dynamics in the evolutionarily unique models: the planktotrophic development strategy and the energy allocation strategy.

## Dopamine in development: emergence, localization, and functions in bivalve larvae

The phylum Mollusca belongs to the group Trochozoa comprising many phyla from the clade Spiralia [88]. The term Spiralia directly indicates the main feature of this clade: the spiral fragmentation in embryonic development. Species of the subclass Autobranchia, constituting a substantial part of Bivalvia, develop through the planktotrophic, trochophore, and then veliger larva stages. There are also other larval types known for bivalves: the parasitic glochidium, lasidium, or haustorial larvae in the order Unionida [89], as well as the lecithotrophic pericalymma in the subclass Protobranchia [90]. The species that are known to have direct development emerged independently in different evolutionary lineages of bivalves [91–93]. However, in this paper, we highlight the developmental features of the DAergic system of only veliger due to the lack of data on other types of development.

A substantial portion of data on the DAergic system morphology in bivalve larvae was obtained by the FaGlu method, which is specific not only to DA but also to all other CA, adrenaline and noradrenaline. Therefore, when describing the potential DA-containing components of the nervous system, it would be more correct to refer to them as “CAergic”, being a more general term.

The molluscan trochophore is an obovate, ciliated larva that is usually formed on the first day of the animal’s life. At this stage, the larva does not feed because of the underdeveloped digestive system. At the trochophore stage, the nervous system is laid down in the form of paired dorsal and ventral pioneer neurons. Subsequently, a pair of lateral nerve cords appear between them to constitute the basis for the future CNS ganglia. Also, at the trochophore stage, a larval neurostructure is formed in the apical part of the body, or the apical organ (AO), which is of great importance throughout the larval life-history period. All functions of the AO are not fully understood, but it presumably plays a role in the primary chemo- and mechanoreception, the organization of CNS development, and the modulation of larval behavior such as the ascent and settlement processes [94, 95]. It is suggested that the AO is resorbed or undergoes apoptosis by the end of the larval period and upon reaching the juvenile stage [94]. In bivalves, the AO usually consists of several (from three to five) serotonergic cells and one or two FMRFamidergic cells, while no CAergic, including DA-containing, cells have been found in it [96, 97].

**Fig. 4:**
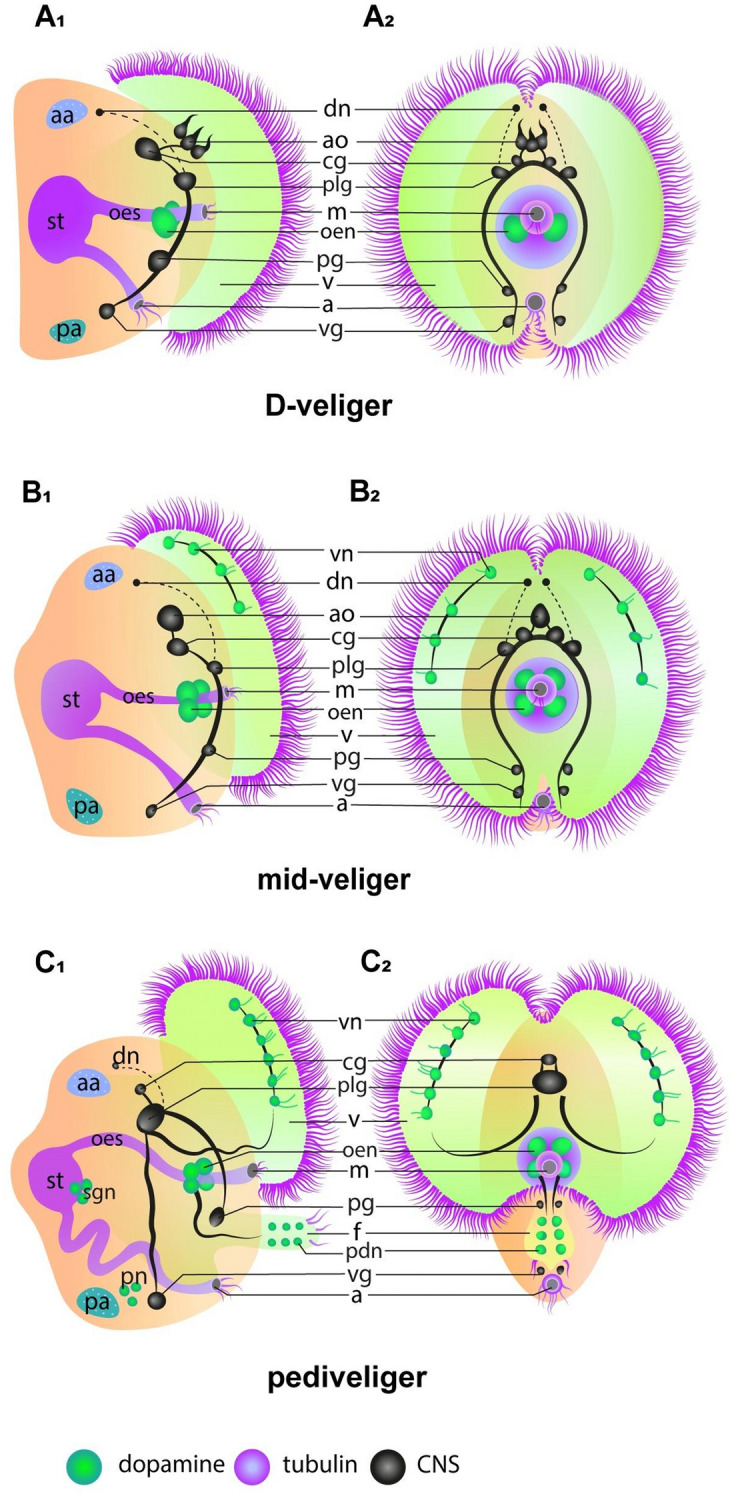
General structure of the nervous system in bivalve larvae: A_1_–A_2_—D-veliger; B_1_–B_2_—mid-veliger; C_1_–C_2_—pediveliger. Color designations: green—DA; magenta—tubulin; black—central nervous system (CNS). Letter designations: a—anus; aa—anterior adductor; ao—apical organ; cg—cerebral ganglia; dn—dorsal neurons; f—foot; m—mouth; oen—oesophagal neurons; oes—oesophagus; pa—posterior adductor; pdn—pedal nerves; pg—pedal ganglia; plg—pleural ganglia; pn—pallial neurons; sgn—stomatogastric neurons; st—stomach; v—velum; vg—visceral ganglia; vn—velum neurons

When the larva develops a bivalve shell, a velum, and a digestive system, it becomes a veliger. In the early stages, the veliger has a D-shaped shell, for which this life-history stage is also referred to as D-veliger [96, 98]. The velum consists of paired, lobe-shaped outgrowths fringed by cilia. It is formed by stretching the episphere along with the prototroch and metatroch and is present throughout the larval period until settlement, followed by its resorption [96, 98]. With the velum, the larva can hover in the water column, capture food particles from the environment, and convey them into the mouth, thus, leading a planktotrophic lifestyle. At this stage, the larva begins to feed, and the first pair of CAergic neurons appears in the anterior esophagus near the mouth (Fig. 4, A1–A2) [96, 97]. Later on, at the mid-veliger stage, a second pair of perioral neurons is formed, and chains of 3–4 catecholamine nerve cells appear in the velum lobes (Fig. 4, B1–B2) [96, 97]. Then, at the pediveliger stage, the larva develops the foot, an organ with which it can move on the substrate. In this period, the velum is retained, which allows the pediveliger to combine two modes of movements: to swim in the water column using the velum or to crawl along the substrate using the foot. By the pediveliger stage, the larva’s CA nervous system grows more complex: neurons are found in the foot, on the stomach wall, and in the posterior adductor region (Fig. 4, C1–C2) [96, 97]. Pediveliger is the final larval life-history stage when the larva is actively searching for a substrate to settle and subsequently metamorphose into a juvenile. During the metamorphosis, the pediveliger loses a number of larval organs such as the velum and the AO and acquires a number of definitive ones: the gills with siphons and the circulatory system [96, 97].

It is currently unknown whether the larval CAergic neurons are lost subsequently during metamorphosis and are replaced by definitive neurons at the adult stage or the anlagen formed in the early developmental stages then become the definitive nervous system components. This issue can be addressed by studying the metamorphosis process, i.e., the transition from the pediveliger to the juvenile stages. The major challenges are the difficulty of rearing bivalve larvae because of their high mortality rate and the short time of the metamorphosis process which is hard to detect. Thus, any data on the fate of the DAergic components of the bivalve nervous system during metamorphosis will be of great value. Such information will help link the larval and definitive morphologies of the DAergic system.

Thus, DA, being one of the most important biogenic amines, regulates the growth, morphology, and behavior of the larval stages in bivalves. During development, various environmental factors change the level of endogenous DA. Ontogenic variations in the DA content in larvae have been reported for *Pecten maximus* [99]. An assumption has been made that an increased CA level enhances the larva’s ability to metamorphose and disperse.

Besides morphological changes, bivalve larvae exhibit complex behaviors, e.g., swimming and dispersal. Both behavior and morphology undergo profound changes at certain developmental stages. The DAergic effector pathways in bivalve larvae have been described through experiments on *M. edulis* and *C. gigas*. DA and CA are known to induce dispersal and metamorphosis in *C. gigas* [100]. Molecular pharmacological approaches allow elucidating the role that DA receptors play in the larva’s response to CA exposure. In particular, the first orthologue of DA receptors has been described from bivalves, which provides insight into the evolution of DA receptors [48]. In *M. edulis*, CA modulates swimming activity and response to light brightness [8]. These results contribute to understanding the role of CA in the development of bivalve larvae and provide models for a broader study of DA functions in protozoans.

Of particular interest is the finding that such parameters as the timing of onset of critical development stages, the degree of survival, and the motor activity of larvae are flexible and can be adjusted depending on variations in the environmental factors: abundance and species of algae used as food, salinity, temperature, pH, presence of toxicants, and oxygen concentration [94, 101–103]. Being sessile filter-feeders, bivalves settle onto the substrate during the larval development and metamorphose into juveniles. Missed opportunities for dispersal can lead to a reduction in bivalve populations. Moreover, bivalves often suffer from toxic algal blooms [104]. Under laboratory conditions, control larvae developed normally through all the stages, from trochophore to spat. However, concentrations of CO_2_ similar to those during algal blooms, as well as an increased CO_2_ level, significantly affected the timing of metamorphosis, which caused over-development of larvae without metamorphosis [105]. The mechanisms underlying the perception and response to environmental signals are crucial for larvae to successfully cope with changes in habitat conditions.

DA and DAergic receptors have been suggested as key factors regulating the timing of onset of the critical stages [8, 100, 106]. Such a plasticity of larva’s response to DA has potential implication for understanding bivalve population dynamics and spatial distribution. In general, DA, CA, and CAergic receptors play an important role in providing the successful development of larvae.

## Serotonin–dopamine interaction in larval behavior

5-HT and DA are functionally opposite to each other at all stages of bivalve development. In addition to the effects on the beating rate of lateral cilia of the gills in adults, similar antagonistic interactions occur during the larval life-history stage. Incubation of veliger larvae in seawater with 5-HT concentrations from 10^–7^ M to 10^–6^ M led to an increase in motor activity manifested as more active swimming throughout the volume compared to control group and as abnormal rotating movement exhibited by few larvae; incubation with DA concentrations from 10^–4^ M down to 3 × 10^–6^ M induced the velum to contract, the shell valves to close, and larvae to remain on the bottom [8]. The same exposure conditions influenced not only physical but also feeding activity. An increase in DA concentration led to a decrease in the larval ingestion rate until complete suppression of feeding. 5-HT concentrations from 10^–7^ M to 10^–6^ M led to an increase in the larval ingestion rate, but higher concentrations induced a marked decline in feeding activity [8].

Metamorphosis is a crucially important period in the bivalve ontogeny when the organism develops from the larval to adult stages. There are assumptions that the adrenergic and DAergic pathways, with the adrenergic and DAergic receptors being their key components, are responsible for such larval processes as settlement and metamorphosis [107]. The exposure of competent larvae to L-DOPA and DA, as well as to adrenaline and noradrenaline, led to acceleration of such metamorphosis processes as attachment to the substrate, loss of the larval organs (velum), and growth of the definitive ones (prodissoconch and gills) [108]. For a long time, the main focus was only on this pathway, but, as it became clear subsequently, there could be a serotonergic pathway, since 5-HT exposure also caused acceleration of metamorphosis [109, 110]. It is relevant to note that blocking of glutamate-specific NMDA receptors led to an increase in the larval metamorphosis rate as well [106]. The process of transition to the definitive, adult stage in bivalves is an extremely important, complex, and, sometimes, even species-specific process regulated by numerous mechanisms and neurotransmitters and is sensitive to a variety of environmental parameters, which opens up a vast field for research.

**Fig. 5:**
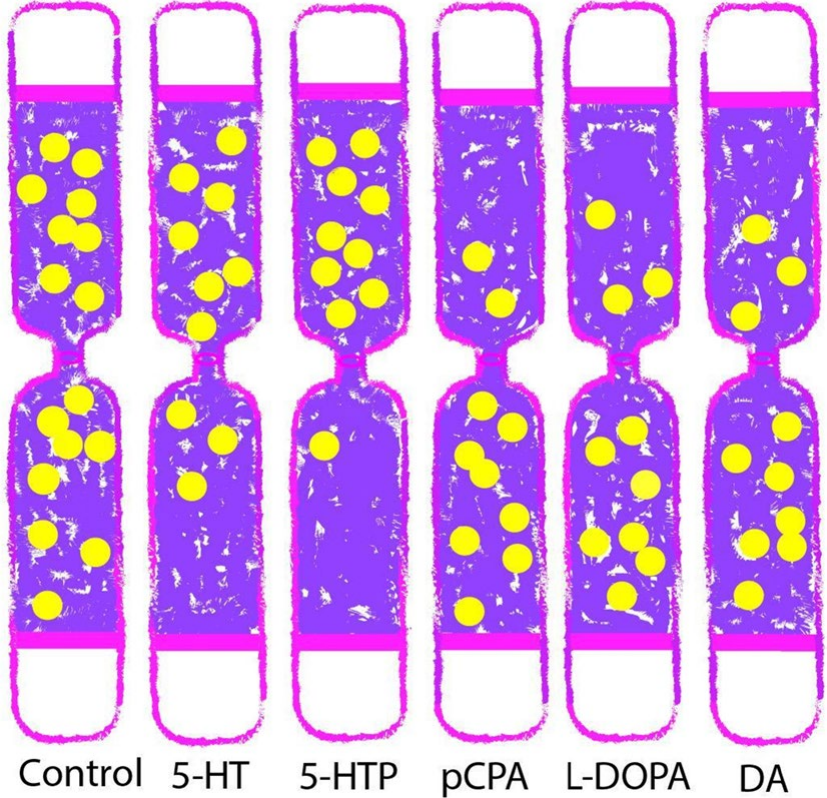
Diagram of an experiment with incubation of bivalve mollusk larvae with substances that modulate serotoninergic (5-HT—serotonin; 5-HTP—5-hydroxytryptophan; pCPA—para-chlorophenylalanine) and dopaminergic activity (DA – dopamine; L-DOPA—L-dihydroxyphenylalanine). As a result, the motor activity of larvae changed, which was manifested as their movement in the volume of the medium either to the upper (activation) or to the lower (inhibition) parts

According to our unpublished data, incubation of bivalve larvae at the D-veliger stage with substances that affect the serotonergic and DAergic systems led to changes in the larval motor activity (Fig. 5). An increase in the concentration of 5-HTand its metabolic precursor, 5-hydroxytryptophan (5-HTP), to 10^–7^ M caused an overall increase in the activity of larvae, and, as a result, they ascended to the upper part of the medium volume. Inhibition of 5-HT synthesis by 10^–7^ M pCPA (para-chlorophenylalanine) caused a decrease in the larval motor activity. After D-veligers were incubated with DA and L-DOPA, they showed lower motor activity, with most of them found in the lower part of the medium volume (Nikishchenko, personal data).

## Dopamine in Bivalvia from an evolutionary aspects

The evolutionary history of DA has been traced back to either free-living organisms that utilized DA as a signaling molecule or organisms that metabolized DA from melanin [111–113]. A comparison of the phylogenetic aspects of the DA systems has shown that some (but not all) bivalves have a developmental type of DA system that seems to be evolutionarily young and shared by them [63, 114]. DAergic cells are found in both bivalves and gastropods but presumably perform different functions [63, 115, 116]. In cephalopods, the systems have changed independently, although they might be similar initially [63, 114, 117]. In polyplacophorans, the buccal (head) ganglia contain two pairs of DAergic neurons. One pair of large neurons projects to the lateral aspect of the ganglion, and the other projects ventrally and dorsally, innervating areas of several neuropil modules that comprise the sensory, motor, and interneuron pathways [117, 118]. These neurons are likely involved in feeding-related behaviors that are controlled by this mechanosensitive and chemosensitive ganglion.

The main DAergic pathway has remained unchanged across various animal phyla. In bivalves, as in other organisms, there is one tyrosine hydroxylase that catalyzes the rate-limiting reaction. However, bivalves presumably differ in how the DA efflux is carried out. In bivalves, two SLC22 transporters have been identified, of which one likely transports CA and the other may transport amino acids [119]. Some bivalves have a transport protein similar to those currently considered DA transporters in other animals. *N*-acetylation, catalyzed by *N*-acetyltransferase, seems to be the main pathway for terminating the DA action in bivalves [120, 121]. There are also two acyl-CoA synthetases, of which one has been shown to terminate the CA action in insects and crustaceans [121, 122]. In general, competitive and non-competitive DA antagonists are evolutionary conserved across the animal phyla. Drugs that regulate DA levels in tissues such as Levodopa and D2-like agonists are widely used to improve the motor symptoms of Parkinson’s disease. Environmentally, death of dopaminergic neurons can be induced by acute exposure to manganese, 6-hydroxidopamine, and MPTP [55]. Consideration of DA-related mechanisms and functions from an evolutionary perspective significantly contributes to understanding how DA and the modulatory mechanisms have changed, adapted their function(s), and how these changes and adaptations have influenced behavior.

## Dopamine and climate change: implications for bivalve populations

Bivalve populations have existed for at least 500 Ma, survived five mass extinctions, and then successfully radiated and adapted to a wide variety of niches and environments. This success presumably stems from a suite of “bivalve-specific” attributes and a robust life-history strategy involving shifts between the free-living modes driven by climate and seasonal conditions and the sediment-dwelling, quasi-quiescent adult stages [123, 124]. Recent studies highlight the significant changes in bivalves’ reproductive success and population sustainability in the face of changing climate. On the one hand, the projection models of future climate change scenarios suggest that naturally high CO_2_ and temperature levels will negatively affect bivalve calcification and growth due to the inhibition of carbonic anhydrase, one of the major functional enzymes in shell formation [125, 126]. On the other hand, progression of harmful algal blooms may impair filtration and cause mortality in bivalve populations. Bivalves’ exposure to the direct negative effects of the projected marine climate change may alter their physiological functions, often through interactions of multiple drivers along the food chain [127].

## Future trends in dopamine research in Bivalvia

Some studies on the role of DA could be facilitated by technological advances such as novel in vivo imaging tools that are rarely used for bivalves but may allow mapping DAergic neurons in living animals [128, 129]. By combining genetic or pharmacological manipulations of the DAergic system with behavioral analyses, it becomes possible to elucidate the role of DA in learning and memory in bivalves, similarly to studies on gastropods and cephalopods [130–132]. Neuroecological approaches related to DA can also be applied to bivalves. Bioenergetic models may be combined with measurements of natural environmental stress factors such as hypoxia and phytoplankton blooms to better understand their effects on DA-mediated behavior [133]. There are also major issues concerning the DA system of bivalves that need to be addressed before embarking on more applied research [134].

DA can be used as a model biogenic amine in comparative studies of behavior in bivalves, gastropods, cephalopods, and large brachiopods. Bivalves may be a representative group for considering the evolutionary transition from tonic to phase release of DA, which is reported to have occurred in several Spiralia groups [115]. As filter-feeders, bivalves can be suitable models for studying the effects that changes in the food web exert on the DA-mediated behavior. Due to the importance of bivalves in the biogeochemistry of sediments, data on their behavior may contribute to the study of the role of DA in a broader ecosystem context. Efforts are also needed to apply the knowledge of DA in bivalves’ behavior, gained through basic research, to the current programs for their conservation. For example, the knowledge about the role of DA in encoding environmental signals that promote larval dispersal and about the effects of pollutants on these signals can be used to improve habitat conditions in coastal areas. Thus, future studies of DA in bivalves may contribute to understanding the diverse roles of DA in the life-history evolution.

## Conclusions

DA in bivalves has been a subject of numerous studies for several decades. As a result, a vast array of basic data has been collected concerning the morphology, physiology, and the role of DA as an inhibitor of ciliary beating and feeding activities and a regulator of larval behavior. Of particular interest are the antagonistic interactions of DA with 5-HT, with the latter being also involved in the regulation of the above-considered functions. Nevertheless, many issues as to the DAergic system in a vast number of marine and most freshwater species and its manifestation in various types of larvae still remain unaddressed. Since DA is important in feeding regulation, a detailed study of this phenomenon can provide tools to enhance survival and growth rates of bivalves in various environmental conditions and, especially, in aquaculture.

## Data Availability

Not applicable.
